# Spanish residents’ experiences of care during the first wave of the COVID-19 syndemic: a photo-elicitation study

**DOI:** 10.1080/17482631.2023.2172798

**Published:** 2023-02-13

**Authors:** Laura Medina-Perucha, Constanza Jacques-Aviñó, Tomàs López-Jiménez, Catuxa Maiz, Anna Berenguera

**Affiliations:** a Unitat Transversal de Recerca, Fundació Institut Universitari per a la recerca a l’Atenció Primària de Salut Jordi Gol i Gurina (IDIAPJGol), Barcelona, Spain; b Universitat Autónoma de Barcelona Bellaterra, Cerdanyola del Vallès, Spain; c Network for Research on Chronicity, Primary Care, and Health Promotion (RICAPPS), Spain; d Departament d’Infermeria, Universitat de Girona, Girona, Spain

**Keywords:** Care work, COVID-19, lockdown, gender, photo-elicitation, self-care, collective care, Spain

## Abstract

**Purpose:**

The main aim of this research was to explore experiences of care during the lockdown of the first wave of COVID-19 syndemic in Spain

**Methods:**

This is a qualitative and explorative study using self-photo-elicitation as a data collection method. Fifteen participants (Twelve women and three men) shared 25 photographs and one video between the June 18 and August, 2020. Participants’ photographs and texts were collected online. Data were analysed based on Thematic Analysis.

**Results:**

Three emerging categories were constructed: 1) the deconstruction of care: self-care and collective care 2) the crisis of care and gendered care, 2) beyond anthropocentrism: animalism and ecology. Findings indicate the need to understand “care” in terms of social reproduction, including self-care, care towards other humans and non-human animals, and collective care. Also, the need to care for planetary health and to be in contact with nature as a form of self-care and social care.

**Conclusions:**

Care in a period of social and health crisis puts human relationships and also non-human life at the centre. Care requires adopting taking an ecological one-health perspective.

## Introduction

Research and sociopolitical debates around the impact of the COVID-19 pandemic have mostly focused on morbidity and mortality. However, the COVID-19 pandemic has also forced us to critically look into our systems’ health and the determinants of health (Harish, 2021). Some academics are already focusing their work on understanding how systemic social and political structures are connected to the COVID-19 pandemic (Chatzisakis et al., 2020; MacLeavy, 2020; Malherbe, 2020). Social health inequities in the context of the COVID-19 pandemic are already well-documented (Fisher et al., 2020; Jacques-Aviñó et al., 2020), suggesting the need for *social medicine* (Ashton, 2006; Trout et al., 2018) and other social sciences to respond to what can be considered a *social disease* (Trout & Kleinman, 2020). Also, some authors are already referring to the COVID-19 pandemic as a *syndemic* (Harish, 2021; Horton, 2020), a concept introduced by medical anthropologists to conceptualize ill-health in complex pandemics by understanding the intersection between socio-environmental and biological factors (Singer et al., 2020). This is the term that will be used throughout this paper, to acknowledge and consider socio-environmental that are linked to the development and consequences of COVID19. Also, as we understand the need to include social, structural and environmental factors in COVID-19 policies.

The current COVID-19 syndemic has been established as a *crisis of care* (Chatzisakis et al., 2020), which inevitably links back to the crisis of care previously theorized as the result of neoliberal politics (Fraser, 2016; Pérez Orozco, 2006). This political model implies the prioritization of monetized economic powers and markets, considering people as mere tools to sustain systemic structures (Pérez Orozco, 2006). The crisis of care refers to the challenges that neoliberal societies face to ensure *social reproduction*, a term that encompasses self-care and care for others (childcare, eldercare and healthcare) while maintaining physical spaces and organizing required resources (cleaning, shopping, repairing), and human reproduction (having and raising children) (Arruzza, 2016; Hester, 2018). It is both a political and ethical crisis (Malherbe, 2020) that comes with the peril of deepening intersecting oppressions towards social agents (mostly women) (Cohen & van der Meulen Rodgers, 2021) who are made socially and politically responsible for ensuring social reproduction to sustain the monetized spheres of our economies (European Commission, 2021). Studies conducted during the COVID-19 syndemic have shown that this work overload has had impacts on mental health, both in terms of work-life balance and the “mental burden” of caregiving responsibility (Fisher et al., 2020; Jacques-Aviñó et al., 2020, 2022; Matulevicius et al., 2021). Thus, we cannot afford to neglect it if we want to ensure individual and collective survival. Especially, if we understand the experiences of care (referring to the physical or symbolic elements that allow people to survive in society) as a framework for understanding how to act in a crisis, as it encompasses all the heterogeneous practices that constitute the maintenance and repair of something (Anigstein et al., 2021).

Therefore, the crisis of care has led to a destabilization of the previous redistributions of social and monetized reproduction (Malherbe, 2020; Pérez Orozco, 2006). In fact, the COVID-19 syndemic has led to a reorganization of labour due to the loss of job opportunities, digital work and work-from-home policies (MacLeavy, 2020). These new realities may have a higher negative impact on women, explained by the feminization of paid and unpaid care, higher unemployment and employment insecurity, and the pay associated with a gender-segregated workforce (MacLeavy, 2020). According to research carried out with heterosexual couples during 2021, women reported having fewer physical and temporal space limits to manage productive and reproductive work time than men. While men reported doing care work once they have finished their productive tasks (Chauhan, 2022). This can be partly explained by women’s web of culturally-assigned responsibilities and role both in non-monetized and monetized activities. In fact, the closure of school and daycare centres has had particularly affected impacted female carers (Alon et al., 2020). In addition, other research has shown that some women would feel extraordinarily vulnerable if they were faced with closed schools in the future, knowing that the need to reduce productive working hours to care for dependents would be an aggravating factor for them (Soubelet-Fagoaga et al., 2021).

Thus, we are living an unprecedented situation for today's society and so we need to understand how the population conceptualizes and experiences care in the context of the current syndemic (Chatzisakis et al., 2020). The current study was initiated to respond to the need to focus on “care” as a central element in the analysis of the impact of the COVID-19 syndemic in Spain. Especially considering the strict lockdown that occurred in Spain during the first wave of COVID-19 and the existing (gendered) crisis of care in our context prior to the syndemic (Río Lozano M et al., 2021). Throughout this research we intend to initiate, or rather continue, the conversation on the need to place care at the centre of the public and private response to the COVID-19 syndemic. Also, to our knowledge, there is a gap qualitative studies conducted during this period, especially from non-verbal technique. Therefore, our main aim was to explore experiences of care during the lockdown of the first wave of the COVID-19 syndemic in Spain. This aim was formulated to address the following research questions: How do the images represent the experiences of care in lockdown during the first wave of COVID-19 in Spain? What relationship do people make between the image and the text about their experiences of care during the first COVID-19 lockdown? This study is part of a larger mixed-methods project that aims at evaluating the psychosocial impact of COVID-19 syndemic in Spain, taking a gender-based approach (Jacques-Aviñó et al., 2020).

## Methods

The work presented in this article is an exploratory qualitative study using photo-elicitation (Collier, 1957; Harper, 2002) as a method for data collection. Our study has been conducted using a gender-based perspective and a critical paradigm. The choice of photo-elicitation was justified by the possibility of capturing expressions of experience that could not be apprehended in any other way. Using photographs could be an advantage to evoke *“deeper elements of human consciousness”* (Harper, 2002), compared to the analysis of words or narratives. The use of photo-elicitation has also been reported to enrich the research process. This is especially relevant when combined with narrative data. Also, when participants are able to take photographs themselves, they can metaphorically represent what is most important to them and thus guide researchers on what to focus on. Photo-elicitation is helpful to promote rapport and enable researchers to understand participants’ perspectives and narratives through their own lens (Berenguera et al., 2017). This was useful as data were collected under strict lockdown and all research data had to be collected virtually. Besides, photo-elicitation could allow the emergence of unexpected topics, which we considered highly relevant given the lack of evidence on the impact of COVID-19 syndemic at the time of data collection, and the need to conduct exploratory research on this topic. Photo-elicitation could also aid data analysis and interpretation by including participants’ own photographs and narratives (Collier, 1957; Harper, 2002; Meo, 2010). This method was also deemed appropriate given that conducting face-to-face research was not possible due to the COVID-19 public health measures at the time of data collection.

The quality and rigour of our study was assessed by using the Guba & Lincoln’s criteria, ensuring the credibility, transferability, dependability and confirmability of the research (Guba & Lincoln, 1994). We used the Standards for Reporting Qualitative Research (SRQR) tool to ensure reporting standards for qualitative studies (O’brien et al., 2014).

## Participants and recruitment strategy

Participants were adults (>18 years old) who self-identified as “carers” and resided in Spain during the first wave of the COVID-19 syndemic. Although the researchers planned a selective and purposive sampling, convenience sampling was followed due to limitations during recruitment. Participants were recruited based on their participation in a previous survey-based study (Jacques-Aviñó et al., 2020) on the psychosocial impact of the COVID-19 syndemic in Spain. Those participants who had identified themselves as “caregivers for others” (minors and other dependent people) in the survey were invited by email to take part in the photo-elicitation study. The invitation email was sent three times to 392 people in total. Fifteen participants, 12 women and three men aged 22–68, took part in the study (see Table 1 for participants’ socio-demographic data). The team considered sufficiency and saturation criteria to determine the sample size. A few potential participants declined taking part as they preferred not to share “personal” images or did not have time to engage in the study.

**Table I.** Participant sociodemographic characteristics.

Participant ID	Sex	Age	Country of birth	Completed education	Employment status
P1	W	38	Argentina	University	Unemployed, receiving unemployment benefits
P2	W	65	Spain	University	Self-employed
P3	W	51	Spain	Technical education	Other/Unknown
P4	W	49	Spain	A-level	Full-time worker
P5	M	22	Spain	Secondary school	Student
P6	M	48	Spain	Technical education	Self-employed
P7	W	44	Spain	University	Full-time worker
P8	W	35	Spain	University	Full-time worker
P9	M	44	Spain	University	Full-time worker
P10	W	45	Argentina	University	Full-time worker
P11	W	68	Spain	University	Retired, receiving State Pension
P12	W	53	Spain	University	Full-time worker
P13	W	65	Bolivia	Technical education	Retired, receiving State Pension
P14	W	23	Spain	University	Full-time worker
P15	W	48	Spain	University	Full-time worker

## Data collection

Data were collected through photo-elicitation methods (Collier, 1957; Harper, 2002). We specifically used a method called photo-self-elicitation method (Harper, 2002) or participant photography (Miller & Happell, 2006) which consisted in asking participants to share a photograph based on the following instruction: “*Take a photograph that represents your experience caring for others (children or adolescents, people with disabilities, or other people) during lockdown”*. Besides, participants were asked to give meaning to their photograph by choosing a title for the photograph and including a short text explaining what the photograph represented for them.

Participants shared 25 photographs and 1 video overall. This was because three participants sent more than one photo; one of them also included a video. Data were collected by email from 18 June 2020 to 7 August 2020.

## Data analysis

Data were analysed based on Thematic Analysis (Berenguera et al., 2017; Braun & Clarke, 2006). Although there are no particularly prescribed methods for analysing and interpreting photo elicitation research, Thematic Analysis (Braun & Clarke, 2006) is one of the commonly applied methods to ensure rigour in data analysis in photo elicitation studies (Murray & Nash, 2017). In this study all five authors participated in the analysis process. First, the researchers independently described and analysed all photographs and identified themes in photographs’ titles and descriptions provided the participants. Visual and narrative elements for each participant were combined, so that themes and sub-themes could be identified for each case. This was an inductive and reflexive process that was later shared throughout several team meetings. These discussions were useful to triangulate the analysis and to identify initial pre-analytic ideas and for the first author to construct an initial thematic framework, based on the independent analysis conducted by the researchers. Thus, themes and sub-themes were identified across participants’ data. A final joint analysis and discussion session was organized with all researchers to discuss final thematic framework.

Individual and collective reflexivity was an important tool used by the research team throughout the study, and especially during data analysis. For instance, analysis meetings included discussions around how the researchers understood “care”, what their perspective was on what might be considered a rural context, or the researchers’ deconstructions on anthropological privileges and the focus on animalism. These discussions were highly valuable in situating the researchers’ perspective on the research and the collected data. Hence, these collective insights were part of the analytical process.

## Ethical considerations

All participants were given information about the photo-elicitation study and were requested to give their written consent prior to their participation. Although parental/legal guardian consent was solicited when photographs included minors or people with disabilities, identifiable photographs will not be included in this article for ethical reasons. Photographs of those participants who refused to make their photographs public will not be disseminated either. This study was granted ethical approval by the Institut de Recerca en Atenció

Primària Jordi Gol i Gurina (IDIAPJGol) Ethical Committee on 4th of April 2020 (reference 20/063-PCV).

## Results

Three themes were identified: (1) The deconstruction of care: self-care and collective care; (2) The crisis of care and gendered care; and (3) Beyond anthropocentrism: animalism and ecology.

## The deconstruction of care: self-care and collective care

Although the researchers explicitly asked participants to share their experiences of caring for others, some of them discussed experiences of self-care and collective care. In some cases, having more time for oneself during lockdown had become an opportunity to reflect on (and question) one’s life and to be grateful for the new insights that this period of life was bringing.

“But I had time to cook, look at the scenery, listen to and feed the birds that came to my terrace. Knowing that the previous life, was not a life” (P3)

**Figure 1.**
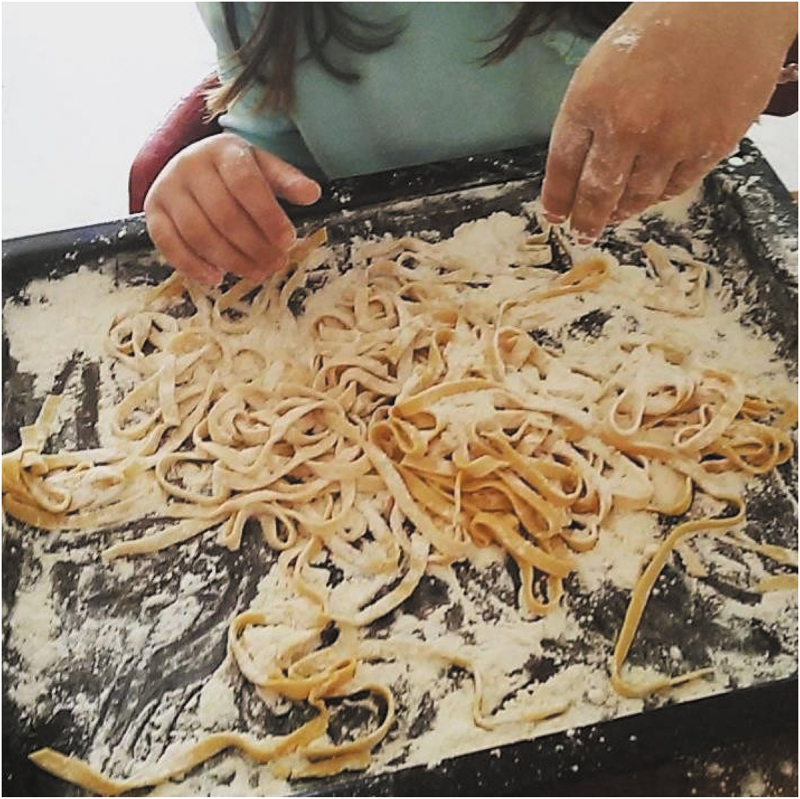
“Cocinando con alegría” (*Cooking with joy*) (P10).

Caring for others was often framed as a form of self-care, representing self-care through fulfilling one’s needs for social interaction, love and belonging, and freedom (Figure 1). However, self-care often seemed to be relegated to other people’s needs, especially among participants who were main carers for children or cared for someone with a disability. Besides, some participants mentioned situations in which they had the opportunity to interact and establish relationships of care with other people, situations that were rather unusual or non-existent prior to the syndemic. It seems then that, despite the isolation, the syndemic had brought unexpected opportunities to connect with and care for other people. Somehow, care seemed to turn into being more collective. Receiving care appeared to be more valued at critical times, compared to before the COVID-19 syndemic.

Seven in the evening in the emergency room after nine hours of sweat, running around, two gowns, two gloves, two glasses without being able to touch our face and my supervisor arrives with cold drinks that, one of many organizations that expressed solidarity during lockdown, has donated to us. I would have never thought that I would cry over a cold Coke; cry out of thirst, cry of feeling so much neediness, cry of emotion after the surprise that someone cares for me (P12)

“Family moments together, reinventing activities with neighbours, who we barely knew until that time” (P15)

On the other hand, some participants explained how they had experienced a deep lack of care during lockdown. They shared moments of despair and helplessness when trying to seek medical assistance, being isolated and/or left without a support system to ensure social reproduction. Their needs were represented in the spaces and objects photographed.

“I am sending the image of the portrait called The Scream of Munch. It represents the anguish because of the loneliness. There was no one on the other side. The doctors did not respond” (P11)

“I am sending a few photos of the hard lockdown, trying to entertain my grandchildren, while my daughters worked at the Pharmacy. It was really very difficult … ” (13)

**Figure 2.**
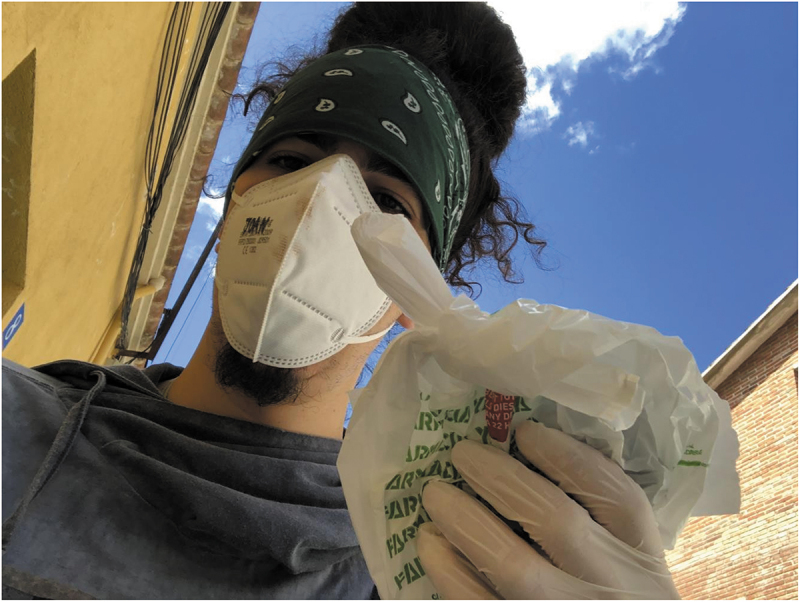
“Apoyo mútuo” (*Mutual support*) (P5).

Related to the previous point, one participant explained that new mutual support networks were created during lockdown. The aim of these networks was to provide assistance to those who could not otherwise obtain it (eg. Elderly or chronically ill people or those living with a chronic health condition). This participant called for the need of collective care, suggesting that institutions were not able to respond to some people’s needs during COVID-19 first lockdown measures (Figure 2).

In the photograph I was delivering medicines to vulnerable people who could not leave their houses. I participate in a mutual support network that has been created in my town with the objective that the people help the people during lockdown (P5)

Participants expressed fear, pain, and hardship, but also of resilience and joy. Some people shared their relief and joy of being reunited with loved ones, and their optimism towards life itself. Thus, despite the adversity of syndemic some participants decided to focus on what they were grateful for, rather than on experiences of grief, sorrow or dread. This could be interpreted in different ways. It could be that participants had not personally and/or consciously experienced emotional distress. Another explanation could be that, at the time of data collection (when lockdown measures were significantly lessened), “positive” feelings of appreciation of day-to-day and social relationships became more powerful.

“The first day we went out in the street after we got cured of Covid 19. [Name], 95 years old and me, her daughter, 65 […] The first day that I saw my grandson after 3 months” (P2)

“This photo represents in part the positives of what this experience has meant … the resilience … the ability human beings have to adapt is fantastic and unpredictable.my dad 86 years old living another way to celebrate his birthday” (P4)

## The crisis of care and gendered care

**Figure 3.**
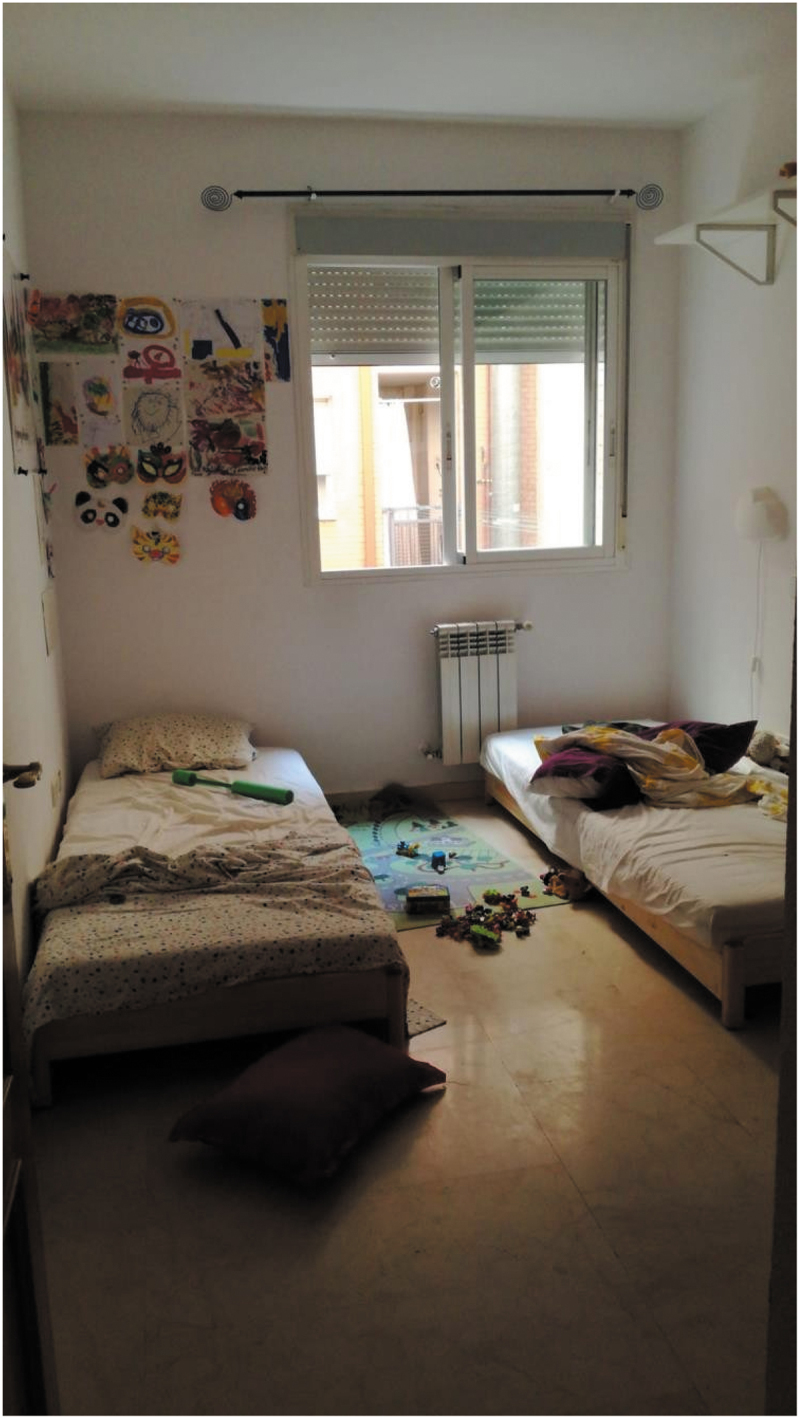
“Su habitación” *(Their room)* (P9).

Care networks were abruptly destabilized once educational centres closed down and some carers (e.g., grandparents) were not available for being considered high-risk populations, mobility restrictions or other responsibilities. Participants shared their experiences of adapting to their new realities, which were sometimes perceived as “chaotic” (P1, P9) but also as opportunities for growth (P4, P7) and connection (P7, P10, P14). The narratives were often a mix between the perceived hardship of the situation and the resistance to change and the need to adjust to living in uncertainty. This chaos that some participants referred to was in some cases represented in the images shared. Some photographs also presented spaces that could be interpreted as signs that participants were living in a situation of social and economic deprivation (e.g., based on furniture in bad condition or the size and light of photographed spaces), (Figure 3).

“This image represents for me the context in which I have lived more than two months locked in with two children who are 7 and 3 years old. It represents the idea that took hold of me regarding the suitability of not trying to tidy up the space permanently, because this was untidy after five minutes by the children. The result consisted of living in a state of permanent untidiness, but at least it did not mean a Sisyphus-type effort … ” (P9)

Participants expressed a sense of (inescapable) responsibility, stoicism and “emotional duty” towards caring. Although caring was sometimes framed as an act of self-care, as discussed above, this could also relate to this stoicism and the powerlessness experienced during the syndemic. This duty to care went sometimes beyond caring for other individuals, and extended to a sense of responsibility to comply with COVID-19 political measures to control the impact of the syndemic.

“The photograph represents the brief time there was sun in the morning that illuminated the house’s balcony in which we studied or just enjoyed the moment, and the responsibility of contributing for everything to go well. Also the acknowledgement of those who, in my opinion, have been the only ones that have complied with lockdown measures with rigour and determination” (P6)

**Figure 4.**
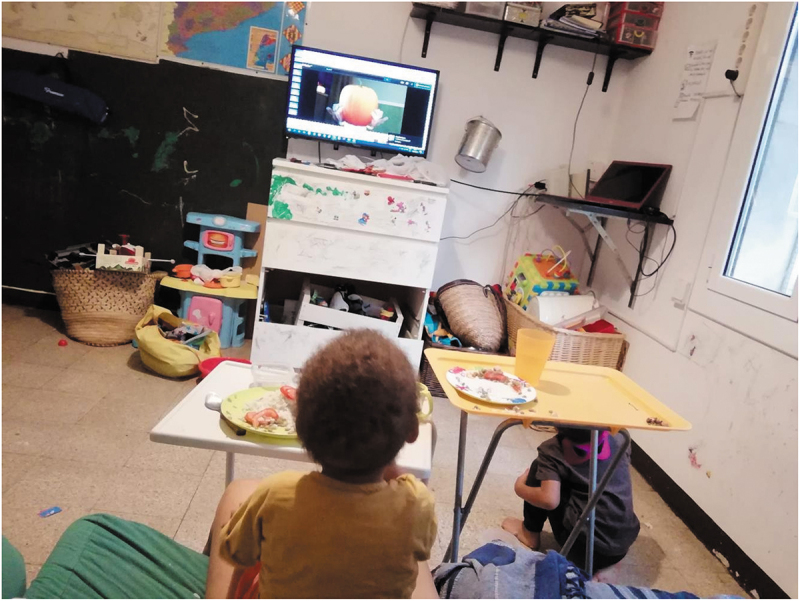
“Caos en casa” *(Chaos at home)* (P1).

It’s worth noting that most participants were women (*N* = 12) and most images and experiences shared were related to social reproduction. Interestingly, it seemed that male carers were more focused on the impact that caring had on them during lockdown (internal focus), while female carers’ photographs and narratives appeared to be rather centred in the experiences of the people they cared for (external focus). Men in our study (*N* = 3) disclosed more frustration while women expressed their tiredness, loneliness and perceived lack of support. While they cared for others they often felt (un)cared for. For one female participant, the closure of borders meant that she was left alone caring for their two children and having to reorganize her day-to-day in order to ensure social reproduction. Although she was sharing her house with other people, she received no help with care responsibilities. Even if we do not know the reasons of this lack of support, it is interesting to consider that physical proximity does not ensure a supportive environment (Figure 4).

“My husband got stranded in Senegal, he is from there and there was no‘real’ possibility of coming back and I was alone all the time (…) I share a flat with 2 people but they were inexistent, pretty much” (P1)

## Beyond anthropocentrism: animalism and ecology

**Figure 5.**
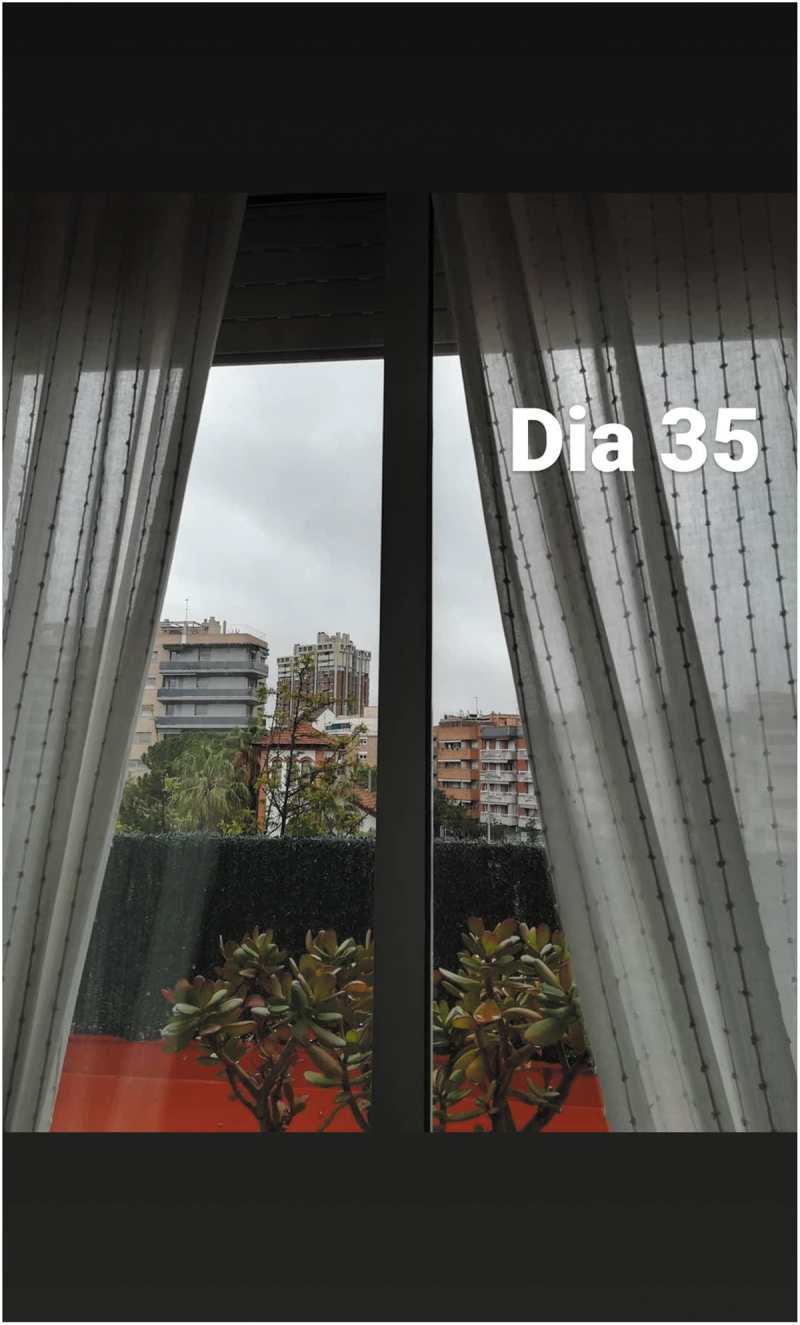
“Tiempo de miedo” (*Time of fear)* (P3).

Some participants expressed the need for a deeper connection with nature and other beings, especially as they had more time in their day-to-day to attend to these needs compared to pre-syndemic times: *“(…) look at the scenery, listen to and feed the birds that came to my terrace (…)” (P3)*. This connection was seen as a way to cope with isolation and was symbolically associated with freedom. Being more in contact with nature and non-human animals was also a way to connect and share time with loved ones. Green and open spaces, even through a window, were photographed in several occasions (Figure 5).

“I was lucky enough to be with my family during lockdown in the countryside where we live and this photo is just of one of the moments when we walked at sunset (…) To me this photo means the start of a new routine that lasted the months we were in lockdown for COVID-19 and that now we maintained once in a while, the walks in nature together, the importance of dedicate some time to share time together and be aware of that” (P14)

“It was helpful to my son to think he had a cat’s life during lockdown: not going outside, but enjoying the ‘terrace’ and the landscape” (P7)

**Figure 6.**
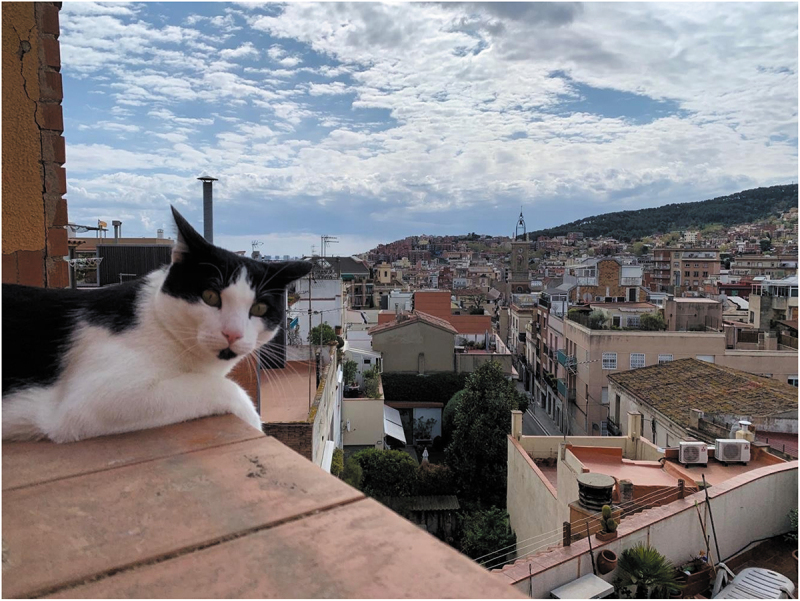
“Sort del gat” (*Lucky we had the cat*) (P7).

Emotional support and care were not only perceived as to be directed to (and received from) other humans, but also other animals such as dogs and cats with whom the participants shared living spaces with during lockdown. There was a feeling of mutual care and gratitude towards animals. Caring for an animal had even created opportunities to meet, get and offer support to others (Figure 6).

“We were also lucky to have our cat! He has been his [participant’ son] best friend, playground buddy and partner these weeks and a great help to make sense of the new routines” (P7)

“Sharing my life with a dog during COVID-19 has meant I have had more emotional support and more structure thanks to my dog Hanna (…) it has also meant I have become friend of several dog-lovers homeless people who live under the building next to my home and that suddenly became visible in the deserted city” (P8)

**Figure 7.**
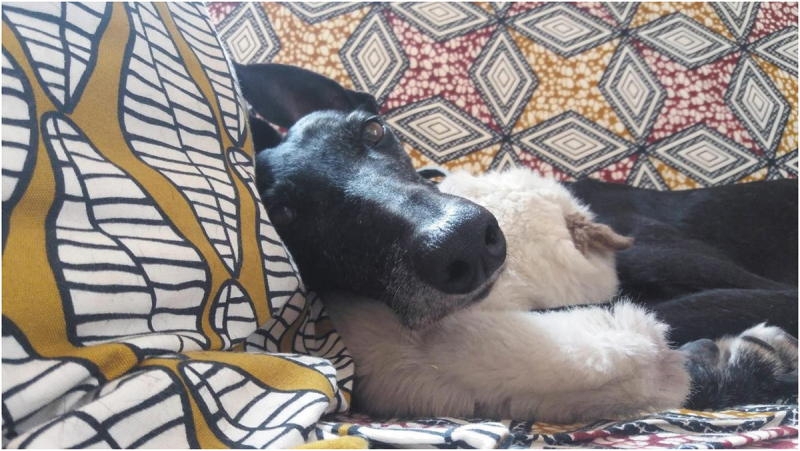
“No olvidando a los que no han podido ser rescatados” (*Not forgetting the ones who could not be rescued*) (P8).

One participant (P8) also perceived dogs as vulnerable to COVID-19 and the effects of the crisis, and expressed anger at the increased abandonment of animals during the beginning of the pandemic due to people’s fear of dogs as vectors of infection. This lack of care, emotional and physical abandonment was seen by her as a symbol of fear and the crisis of care in today’s society. Also, she was worried that violence towards non-human animals might increase, given the impact of the syndemic on people’s emotional health (Figure 7).

“Taking care of a rescued dog during COVID-19 has meant I have worried about the other thousand dogs that have been and are abused everyday—and particularly when people are frustrated and under stress” (P8)

The same participant expressed her frustration regarding public’s disregard for the intersection between planetary and human health “*I have felt defeated and I am really struggling to believe the COVID-19 pandemic will make us understand that human health depends on our environment” (P8)*.

## Discussion

This study contributed to research on social reproduction in the context of the COVID-19 syndemic. Participants’ accounts focused on the need to reorganize their day-to-day lives to ensure social reproduction, and the struggles and impact of doing so after care networks had collapsed. This inevitably links to the crisis of care previously introduced (Chatzisakis et al., 2020; Fraser, 2016; Pérez Orozco, 2006), in which monetized reproduction is prioritized over social reproduction. The COVID-19 syndemic has meant a institutionalization of social reproduction (e.g., through closures of schools and day care centres) (Alon et al., 2020). While social reproduction was previously (at least partly) institutionalized in Spain, it has turned into a merely private matter since the start of the COVID-19 syndemic. Public health and austerity measures due to global financial strains appear to have led to the loss of institutionalized care networks (Bergmann & Wagner, 2021), mostly affecting those who previously shared caring responsibilities and balanced care with paid labour. Based on participants’ narratives, this reorganization of social reproduction has led to a reconceptualization and restructuring of relationships within home units and was associated with emotional struggles. This lack of institutional care was also visible through the challenges that a few participants experienced to access the healthcare system during lockdown (Núñez et al., 2021) and receiving care (Bergmann & Wagner, 2021).

Another important discussion is around the profound impact of the crisis of care on women. Previous research has already indicated the lack of attention to women’s unpaid labour and the costs of fulfilling care roles (Folbre, 2006a), as well as women’s increased vulnerability in the context of health crises, especially among the racialized or migrant populations (Azcona et al., 2020; Cohen & van der Meulen Rodgers, 2021; Doyal, 1996; Jacques-Aviñó et al., Harman, 2016; Jacques-Aviñó et al., 2020, 2022; Sherman, 2020; Smith et al., 2021; Wenham et al., 2020). However, a generalized lack of gendered political and economic responses to the COVID-19 syndemic prevails (Smith et al., 2021). In our research, it appeared that women may feel more responsible for caregiving and the wellbeing of those who they cared for. In contrast, in our study, men’s focus was rather on their own experiences of caring for others, and how caring had an impact on themselves. This suggests that women continue to feel compelled to social reproduction while their own experiences and needs are neglected. Men’s more internal focus could be explained by the lessened social pressures to provide care, and not being as used to balance paid and unpaid labour as women (Jacques-Aviñó et al., 2020). As Smith et al. (2021) argue “not only is COVID-19 a global health crisis, it is also a global gender equity crisis transcending differences in scale, response, and political-economic systems”. Thus, gender and feminist approaches need to be prioritized and incorporated in public health strategies (Kuhlmann, 2009; Smith et al., 2021) so that the value of reproductive care can be socially and economically reassessed. Despite the gender differences identified in our research are supported by evidence and gender-based theory, our findings need to be interpreted with caution given the small number of men included in the study.

In our research highlights the need to acknowledge self-care and collective care. Participants referred to a lack of self-care associated with having to provide care. At the same time other participants shared how being able to dedicate more time to themselves during lockdown was an opportunity to draw their attention inwards and practice self-care. In both scenarios, the importance of self-care during periods of lockdown and, overall, throughout the COVID-19 syndemic needs to be discussed. An increasing amount of evidence on the impact of COVID-19 on mental and emotional health has been published (Jacques-Aviñó et al., 2022; Gloster et al., 2020). Academics worldwide have made a call to prioritize mental health science and public health actions directed to protecting and promoting mental health and its determinants (Holmes et al., 2020). Given that self-care is an important factor to protect and promote health, including mental health (Lucock et al., 2011), we believe it becomes crucial that conceptualizations of care in social, political and research spheres broaden their stance to include self-care. Moreover, research on the associations between self-care and positive health outcomes should be further encouraged (Pilkington & Wieland, 2020). Furthermore, the impact of social reproduction on mental and physical health among informal carers needs to be acknowledged and investigated (Gérain & Zech, 2019).

Housing is a major determinant of health (Bonnefoy, 2007; Krieger, 2001; Vásquez-Vera et al., 2019) and health equity (Swope & Hernández, 2019). Based on participants’ photographs, we could observe structural differences in housing conditions (size, furniture, etc.). Their narratives included housing conditions in terms of the “chaos” and “untidiness” that lockdown and caring responsibilities had brought. It should also be considered that social life in Spain is mostly carried out outdoors and outside one’s home, due to good-weather conditions and culture (Bru-Ronda & Lázaro-Fernández, 2016). Issues associated with suboptimal housing may not have been perceived to be as serious prior to lockdown as they became during lockdown. This could have led participants to share more insights into their living spaces, related to self-care and emotional experience. Considering that social reproduction encompasses the maintenance of physical living spaces (Arruzza, 2016; Hester, 2018), it is relevant to ensure affordable housing, housing quality, residential stability and neighbour opportunity (context) to promote and protect the population’s health (Thomson et al., 2013) and social reproduction. Housing security should encompass tackling energy poverty (Vásquez-Vera et al., 2019), which especially affected vulnerable populations in Spain during the COVID-19 syndemic (Guiteras, 2020) while energy costs continue to rise (FACUA, 2021).

Our research also includes the experience of a young man who was involved in collective care networks. Experiences of collective care through the participation in community-based care networks created during the COVID-19 syndemic are also particularly relevant. Accounting for informal collective care networks can be useful for policy making. It can lead to direct actions towards strengthening, not only healthcare systems, but also community networks (Drury et al., 2019). Reicher and Bauld (2021) have argued how the context of COVID-19 has led to the development of collective resilience, as the syndemic has accentuated a sense of shared identity and collective thinking. Encouraging to rethink the relationship between the institutions and the public, and acknowledging the latter’s power of self-organization and the role of social capital can be key (Wong & Kohler, 2020). However, institutions need to provide enough resources and training to develop and sustain social capital that guarantees social reproduction (Reicher & Bauld, 2021).

Interestingly, some participants shared experiences of caring for non-human animals during lockdown. Sharing life with a non-human animal was framed as a form of social support and as a protective factor to endure lockdown and to create social networks. Attachment and relationships with non-human animals have actually been found to protect and promote mental health (Brooks et al., 2018), even during COVID-19 (Gasteiger et al., 2021; McDonald et al., 2021; Ratschen et al., 2020). Participants also referred to their need to be in contact with nature as a way to alleviate the psychological impact of lockdown, and as a form of self- and social care. This collates with evidence on the positive link between the exposure to nature and mental health outcomes before (Dillman-Hasso, 2021; White et al., 2021) and during the COVID-19 syndemic (Ribeiro et al., 2021). Besides, the need to promote planetary health was mentioned by one participant, as the COVID-19 syndemic was thought to be an outcome of planetary dysbiosis (Hinchliffe et al., 2021). Framing care from an ecological perspective, including non-human animals, contact with nature and planetary health, might be crucial to develop effective public health strategies that also secure universal care (Hinchliffe et al., 2021). The current planetary crisis will require a change in human productive and consumerist practices, as well as socio-economic policies that prioritize sustainable and ecological actions (Guzmán et al., 2021).

This research has limitations. First, we need to acknowledge the potential impact of the digital divide. Despite the team’s effort to reach populations that may have limited access to computers and the Internet, participation may not have been possible for some people. Besides, not being able to contact participants through non-digital tools may have hindered building enough rapport to encourage participation. Despite the researchers’ efforts to motivate more men to participate in the study, only three men participated in our research. This may have limited our gender analysis and interpretation. Another limitation is that even if the use of photo-self-elicitation methods was useful to ensure participation and provides unique and valuable information, data access has been limited to photographs and brief texts sent by participants. However, we had the opportunity to explore innovative research methods such as self-photo-elicitation techniques while still maintaining rigour (Centre for Critical Qualitative Health Research, 2020; Jowett, 2020). It is also important to consider that data collection took place just after the first COVID-19 lockdown in Spain and participants were quite relieved and expressed more positive experiences.

## Conclusions

This research contributes to the visibility of the care crisis in the COVID-19 syndemic in Spain. Based on participants’ experiences of care during the lockdown of the first wave of COVID-19, we promote the need to ensure universal care. This is our capacity and practice to ensure the political, social, material and emotional conditions that allow human and non-human life to prosper. Care needs to be conceptualized in terms of social reproduction, including not only caregiving towards other humans, but also self-care, collective care and care towards (and from) non-human animals. Planetary health and the human need to be in contact with nature should also be considered to ensure social reproduction and promote health. Furthermore, this study is a representation of the resilience of the population to cope with a health and social crisis that required the reorganization of social reproduction. Gender and feminist-based policy strategies need to place social reproduction at the centre and as a determinant of the public’s health, in order to alleviate the crisis of care that the COVID-19 syndemic has deepened. Future research should focus on conducting broader gender analyses of how social reproduction can be ensured during health and social crises, alongside ensuring social equity. Methodological issues, such as equitable gender representation, should be addressed. This should not only include people who identify within a gender binary (i.e., woman/man) but represent a diverse range of gender identities. We believe that this research could support policymaking to ensure social reproduction. Also, to promote human health from a one-health perspective.

## References

[cit0001] Alon, T., Doepke, M., Olmstead Rumsey, J., & Tertilt, M. (2020). The Impact of COVID-19 on gender equality. *Working Paper 26947*. National Bureau of Economic Research. 10.3386/w26947

[cit0002] Anigstein, M. S., Watkins, L., Escobar, F. V., & Osorio-Parraguez, P. (2021). En medio de la crisis sanitaria y la crisis sociopolítica: Cuidados comunitarios y afrontamiento de las consecuencias de la pandemia de la COVID-19 en Santiago de Chile. *Antípoda Revista de antropología Y Arqueología*, 45(45), 53–15. Epub November 16, 2021. 10.7440/antipoda45.2021.03

[cit0003] Arruzza, C. (2016). Functionalist, determinist, reductionist: Social reproduction feminism and its critics. *Science & Society*, 80(1), 9–30. 10.1521/siso.2016.80.1.9

[cit0004] Ashton, J. R. (2006). Virchow misquoted, part-quoted, and the real McCoy. *Journal of Epidemiology and Public Health*, 60 1, 671.

[cit0005] Azcona, G., Bhatt, A., & Davies, S. (2020). Will the pandemic derail hard-won progress on gender equality? Spotlight on gender, COVID-19 and the SDGs. *United Nations Women*. Retrieved from: https://www.unwomen.org/-/media/headquarters/attachments/sections/library/publications/2020/spotlight-on-gender-covid-19-and-the-sdgs-en.pdf?la=en&vs=5013.

[cit0006] Berenguera, A., Fernández de Sanmamed, M. J., Pons Vigués, M., Pujol Ribera, E., Rodríguez Arjona, D., Saura Sanjaume, S., Mahtani Chugani, V., & Cofiño Fernández, R. (2017). *To listen, to observe and to understand. bringing back narrative into the health sciences. contributions of qualitative research*. Institut Universitari d’Investigació en Atenció Primària Jordi Gol (IDIAPJGol).

[cit0007] Bergmann, M., & Wagner, M. (2021). The Impact of COVID-19 on informal caregiving and care receiving across Europe during the first phase of the pandemic. *Frontiers in Public Health*, 9, 673874. 10.3389/fpubh.2021.673874 34222177 PMC8242257

[cit0008] Bonnefoy, X. (2007). Inadequate housing and health: An overview. *International Journal of Environmental Pollution*, 30(3/4), 411–429. 10.1504/IJEP.2007.014819

[cit0009] Braun, V., & Clarke, V. (2006). Using thematic analysis in psychology. *Qualitative Research in Psychology*, 3(2), 77–101. 10.1191/1478088706qp063oa

[cit0010] Brooks, H. L., Rushton, K., Lovell, K., Bee, P., Walker, L., Grant, L., & Rogers, A. (2018). The power of support from companion animals for people living with mental health problems: A systematic review and narrative synthesis of the evidence. *BMC Psychiatry*, 18(311). 10.1186/s12888-018-1613-2 PMC5800290 29402247

[cit0011] Bru-Ronda, C., & Lázaro-Fernández, Y. (2016). Ocio y Cohesión Social a lo largo de la vida. *Revista De Psicología Del Deporte*, 25(2), 73–77.

[cit0012] Centre for Critical Qualitative Health Research. (2020). *COVID-19 Resources Considerations for Conducting Qualitative Health Research During COVID-19 at the University of Toronto*. University of Toronto. Retrieved from: https://ccqhr.utoronto.ca/resources/covid-19

[cit0013] Centre for Critical Qualitative Health Research. (2020). *COVID-19 Resources Considerations for Conducting Qualitative Health Research During COVID-19 at the University of Toronto*. University of Toronto.

[cit0014] Chatzisakis, A., Hakim, A., Littler, J., Rottenberg, C., & Segal, L. (2020). From carewashing to radical care: The discursive explosions of care during Covid-19. *Feminist Media Studies*, 20(6), 889–895. 10.1080/14680777.2020.1781435

[cit0015] Chauhan, P. (2022). “I Have No Room of My Own”: COVID-19 Pandemic and Work-From-Home Through a Gender Lens. *Gender Issues*, 39(4), 507–533. 10.1007/s12147-022-09302-0 35996385 PMC9387412

[cit0016] Cohen, J., & van der Meulen Rodgers, Y. (2021, Aug-Sep). The feminist political economy of Covid-19: Capitalism, women, and work. *Global Public Health*, 16(8–9), 1381–1395. 10.1080/17441692.2021.1920044 33905301

[cit0017] Collier, J. (1957). Photography in Anthropology: A report on two experiments. *American Anthropologist*, 59(5), 843–859. 10.1525/aa.1957.59.5.02a00100

[cit0018] Dillman-Hasso, N. (2021). The nature buffer: The missing link in climate change and mental health research. *Journal of Environmental Studies and Sciences*, 11(4), 696–701. 10.1007/s13412-021-00669-2

[cit0019] Doyal, L. (1996). The politics of women’s health: Setting a global agenda. *International Journal of Health Services: Planning, Administration, Evaluation*, 26(1), 47–65. 10.2190/U7PN-B17E-JQBL-MRG4 8932601

[cit0020] Drury, J., Carter, H., Cocking, C., Ntontis, E., Tekin Guven, S., & Amlôt, R. (2019). Facilitating collective psychosocial resilience in the public in emergencies: Twelve recommendations based on the social identity approach. *Frontiers in Public Health*, 7, 141. 10.3389/fpubh.2019.00141 31214561 PMC6558061

[cit0021] European Commission, W. International Women’s Day 2021: COVID-19 pandemic is a major challenge for gender equality. (2021). Available online at: https://ec.europa.eu/commission/presscorner/detail/en/IP_21_1011.

[cit0022] FACUA. (2021). *92 euros: la factura eléctrica del usuario medio bate todos los récords en la primera quincena de agosto*. FACUA. Retrieved from: https://www.facua.org/es/noticia.php?Id=17147. Accessed on 19th August 2021.

[cit0023] Fisher, J., Languilaire, J. -C., Lawthom, R., Nieuwenhuis, R., Petts, R. J., Runswick-Cole, K., & Yerkes, M. A. (2020). Community, work, and family in times of COVID-19. *Community, Work & Family*, 23(3), 247–252. 10.1080/13668803.2020.1756568

[cit0024] Folbre, N. (2006a). Measuring care: Gender, empowerment, and the care economy. *Journal of Human Development*, 7(2), 183–199. 10.1080/14649880600768512

[cit0025] Folbre, N. (2006b). Nursebots to the rescue? Immigration, automation, and care. *Globalizations*, 3(3), 349–360. 10.1080/14747730600870217

[cit0026] Fraser, N (2016). *Contradictions of capital and care* (vol. 100, series: 99, pp.117). New Left Review.

[cit0027] Gasteiger, N., Vedhara, K., Massey, A., Jia, R., Ayling, K., Chalder, T., Coupland, C., & Broadbent, E. (2021). Depression, anxiety and stress during the COVID-19 pandemic: Results from a New Zealand cohort study on mental well-being. *BMJ Open*, 11(5), e045325. 10.1136/bmjopen-2020-045325 PMC8098295 33941630

[cit0028] Gérain, P., & Zech, E. (2019). Informal Caregiver Burnout? Development of a Theoretical Framework to Understand the Impact of Caregiving. *Frontiers in Psychology*, 10, 1748. 10.3389/fpsyg.2019.01748 31428015 PMC6689954

[cit0029] Gloster, A. T., Lamnisos, D., Lubenko, J., Presti, G., Squatrito, V., Constantinou, M., Nicolaou, C., Papacostas, S., Aydın, G., Chong, Y. Y., Chien, W. T., Cheng, H. Y., Ruiz, F. J., Garcia-Martin, M. B., Obando-Posada, D. P., Segura-Vargas, M. A., Vasiliou, V. S., McHugh, L., Höfer, S. … Francis, J. M. (2020). Impact of COVID-19 pandemic on mental health: An international study. *Plos One*, 15(12), e0244809. 10.1371/journal.pone.0244809 33382859 PMC7774914

[cit0030] Guba, E. G., & Lincoln, Y. S. (1994). Handbook of qualitative research. In N. K. Denzin & Y. S. Lincoln (Eds.), *Competing paradigms in qualitative research* (p. 380). SAGE Publications.

[cit0031] Guiteras, M. (2020). *The right to energy in the EU in times of pandemic. An assessment of the capacity of member states to guarantee it, according to the basic services management model*. Enginyeria Sense Fronteres, Right to Energy Coalition & European Public Service Union.

[cit0032] Guzmán, C. A. F., Aguirre, A. A., Astle, B., Barros, E., Bayles, B., Chimbari, M., El-Abbadi, N., Evert, J., Hackett, F., Howard, C., Jennings, J., Krzyzek, A., LeClair, J., Maric, F., Martin, O., Osano, O., Patz, J., Potter, T., Redvers, N. … Zylstra, M. (2021, May). A framework to guide planetary health education. *Lancet Planet Health*, 5(5), e253–255. 10.1016/S2542-5196(21)00110-8 33894134

[cit0033] Harish, V. (2021). The syndemics of emergency: How COVID-19 demands a holistic view of public health promotion and preparedness. *American Journal of Public Health*, 111(3), 353–354. 10.2105/AJPH.2020.306116 33566666 PMC7893331

[cit0034] Harman, S. (2016). Ebola, gender and conspicuously invisible women in global health governance. *Third World Quarterly*, 37(3), 524–541. 10.1080/01436597.2015.1108827

[cit0035] Harper, D. (2002). Talking about pictures: A case for photo elicitation. *Visual Studies*, 17, 1. 10.1080/14725860220137345

[cit0036] Hester, H. (2018). Care under capitalism: The crisis of “women’s work”. *Progressive Review*, 24(4). 10.1111/newe.12074

[cit0037] Hinchliffe, S., Manderson, L., & Moore, M. (2021). Planetary healthy publics after COVID-19. *Lancet Planet Health*, 5(4), e230–36. 10.1016/S2542-5196(21)00050-4 33838738 PMC8065099

[cit0038] Holmes, E. A., O’Connor, R. C., Perry, V. H., Tracey, I., Wessely, S., Arseneault, L., Ballard, C., Christensen, H., Cohen Silver, R., Everall, I., Ford, T., John, A., Kabir, T., King, K., Madan, I., Michie, S., Przybylski, A. K., Shafran, R. … Bullmore, E. (2020). Multidisciplinary research priorities for the COVID-19 pandemic: A call for action for mental health science. *The Lancet Psychiatry*, 7(6), 547–560. 10.1016/S2215-0366(20)30168-1 32304649 PMC7159850

[cit0039] Horton, R. (2020). Offline: COVID-19 is not a pandemic. *The Lancet*, 396(10255), 10255. Available at. https://www.thelancet.com/journals/lancet/article/PIIS0140-67362032000-6/fulltext 10.1016/S0140-6736(20)32000-6 PMC7515561 32979964

[cit0040] Jacques-Aviñó, C., López-Jiménez, T., Bennett, M., Medina-Perucha, L., León-Gómez, B. B., & Berenguera, A. Self-Reported Anxiety in Spain: A Gendered Approach One Year After the Start of COVID-19 Pandemic. *Front Public Health*. 2022 Jun 16;10:873891. 10.3389/fpubh.2022.873891. 35784235 PMC9244400

[cit0041] Jacques-Aviñó, C., López-Jiménez, T., Medina-Perucha, L., de Bont, J., Gonçalves, A. Q., Duarte-Salles, T., & Berenguera, A. (2020). Gender-based approach on the social impact and mental health in Spain during COVID-19 lockdown: A cross-sectional study. *BMJ Open*, 10(11), e044617. 10.1136/bmjopen-2020-044617 PMC7688440 33234664

[cit0042] Jowett, A. (2020). *Carrying out qualitative research under lockdown—Practical and ethical considerations*. The London School of Economics and Political Science (LSE) Blogs. Retrieved from: https://blogs.lse.ac.uk/impactofsocialsciences/2020/04/20/carrying-out-qualitative-research-under-lockdown-practical-and-ethical-considerations/

[cit0043] Krieger, N. (2001). Theories for social epidemiology in the 21st century: An ecosocial perspective. *International Journal of Epidemiology*, 30(4), 668–677. 10.1093/ije/30.4.668 11511581

[cit0044] Kuhlmann, E. (2009). From Women’s Health to Gender Mainstreaming and Back Again: Linking Feminist Agendas and New Governance in Healthcare. *Current Sociology*, 57(2), 135–154. 10.1177/0011392108099160

[cit0045] Lucock, M., Gillard, S., Adams, K., Simons, L., White, R., & Edwards, C. (2011). Self-care in mental health services: A narrative review. *Health & Social Care in the Community*, 19(6), 602–616. 10.1111/j.1365-2524.2011.01014.x 21749527

[cit0046] MacLeavy, J. (2020). Care work, gender inequality and technological advancement in the age of COVID-19. *Gender, Work & Organization*, 28(1), 138–154. 10.1111/gwao.12534

[cit0047] Malherbe, N. (2020). Community psychology and the crisis of care. *Journal of Community Psychology*, 48(7), 2131–2137. 10.1002/jcop.22427 32789911

[cit0048] Matulevicius, S. A., Kho, K. A., Reisch, J., & Yin, H. (2021 Jun 1). Academic Medicine Faculty Perceptions of Work-Life Balance Before and Since the COVID-19 Pandemic. *JAMA Netw Open*, 4(6), e2113539. 10.1001/jamanetworkopen.2021.13539 34129021 PMC8207238

[cit0049] McDonald, S. E., O’connor, K. E., Matijczak, A., Tomlinson, C. A., Applebaum, J. W., Murphy, J. L., & Zsembik, B. A. (2021). Attachment to Pets Moderates Transitions in Latent Patterns of Mental Health Following the Onset of the COVID-19 Pandemic: Results of a Survey of U.S. Adults. *Animals*, 11(3), 895. 10.3390/ani11030895 33801041 PMC8004029

[cit0050] Meo, A. I. (2010). Picturing students’ habitus: The advantages and limitations of photo-elicitation interviewing in a qualitative study in the city of Buenos Aires. *International Journal of Qualitative Methods*, 9(2), 149–171. 10.1177/160940691000900203

[cit0051] Miller, G., & Happell, B. (2006). Talking about hope: The use of participant photography. *Issues in Mental Health Nursing*, 27(10), 1051–1065. 10.1080/01612840600943697 17050338

[cit0052] Murray, L., & Nash, M. (2017). The challenges of participant photography: A critical reflection on methodology and ethics in two cultural contexts. *Qualitative Health Research*, 27(6), 923–937. 10.1177/1049732316668819 27634295

[cit0053] Núñez, A., Sreeganga, S. D., & Ramaprasad, A. (2021). Access to Healthcare during COVID-19. *International Journal of Environmental Research and Public Health*, 18(6), 2980. 10.3390/ijerph18062980 33799417 PMC7999346

[cit0054] O’brien, B., Harris, I., Beckman, T., Reed, D., & Cook, D. A. (2014). Standards for Reporting Qualitative Research: A Synthesis of Recommendations. *Academic Medicine*, 89(9), 1245–1251. 10.1097/ACM.0000000000000388 24979285

[cit0055] Orozco, P. A. (2006). Amenaza Tormenta: undefined crisis de los cuidados y la reorganización del sistema económico. *Revista de Economía Crítica*, 5(9), 7–37.

[cit0056] Pilkington, K., & Wieland, L. S. Self-care for anxiety and depression: A comparison of evidence from Cochrane reviews and practice to inform decision-making and priority-setting. (2020). *BMC Complementary Medicine and Therapies*, 20(1), 247. PMID: 32778171; PMCID: PMC7418416. 10.1186/s12906-020-03038-8 32778171 PMC7418416

[cit0057] Ratschen, E., Shoesmith, E., Shahab, L., Silva, K., Kale, D., Toner, P., Reeve, C., & Mills, D. S. (2020). Human-animal relationships and interactions during the Covid-19 lockdown phase in the UK: Investigating links with mental health and loneliness. *Plos One*, 15(9), e0239397. 10.1371/journal.pone.0239397 32976500 PMC7518616

[cit0058] Reicher, S., & Bauld, L. (2021). From the “fragile rationalist” to “collective resilience”: What human psychology has taught us about the COVID-19 pandemic and what they COVID-19 pandemic has taught us about human psychology. *The Journal of the Royal College of Physicians of Edinburgh*, 51(1), S12–9. 10.4997/jrcpe.2021.236 34185033

[cit0059] Ribeiro, A. I., Triguero-Mas, M., Santos, C. J., Gómez-Nieto, A., Cole, H., Anguelovski, I., Martins Silva, F., & Baró, F. (2021). Exposure to nature and mental health outcomes during COVID-19 lockdown. A comparison between Portugal and Spain. *Environment International*, 154, 106664. 10.1016/j.envint.2021.106664 34082237 PMC8162907

[cit0060] Río Lozano M, D., García Calvente, M. D. M., & Grupo de alumnado del Diploma de Especialización en Género y Salud de la Escuela Andaluza de Salud Pública-Universidad de Granada. Caregiving and the COVID-19 pandemic from a gender perspective. *Gaceta Sanitaria*. 2021 Nov-Dec;35(6):594–597. Spanish. 10.1016/j.gaceta.2020.05.006. 32553483 PMC7256491

[cit0061] Sherman, K. (2020). Dual pandemics: Coronavirus and gender-based violence. *Think Global Health*. Retrieved from: https://www.thinkglobalhealth.org/article/dual-pandemics-coronavirus-and-gender-based-violence.

[cit0062] Singer, M., Bulled, N., & Ostrach, B. (2020). Whither syndemics?: Trends in syndemics research, a review 2015–2019. *Global Public Health*, 15(7), 943–955. 10.1080/17441692.2020.1724317 32037962

[cit0063] Smith, J., Davies, S. E., Feng, H., Gan, C. C. R., Grépin, K. A., Harman, S., Herten-Crabb, A., Morgan, R., Vandan, N., & Wenham, C. (2021). More than a public health crisis: A feminist political economic analysis of COVID-19. *Global Public Health*, 16(8–9), 1364–1380. 10.1080/17441692.2021.1896765 33705248

[cit0064] Soubelet-Fagoaga, I., Arnoso-Martínez, M., Guerendiain-Gabás, I., Martínez-Moreno, E., & Ortiz, G. (2021 Nov 17). (Tele)work and Care during Lockdown: Labour and Socio-Familial Restructuring in Times of COVID-19. *International Journal of Environmental Research and Public Health*, 18(22), 12087. 10.3390/ijerph182212087 34831843 PMC8620492

[cit0065] Swope, C. B., & Hernández, D. (2019). Housing as a determinant of health equity: A conceptual model. *Social Science & Medicine*, 243, 112571. 10.1016/j.socscimed.2019.112571 31675514 PMC7146083

[cit0066] Thomson, H., Thomas, S., Sellstrom, E., & Petticrew, M. (2013). Housing improvements for health and associated socio-economic outcomes. *Cochrane Database of Systematic Reviews*, 2. CD008657. 10.1002/14651858.CD008657.pub2 PMC12551615 23450585

[cit0067] Trout, L. J., & Kleinman, A. (2020). Covid-19 Requires a Social Medicine Response. *Front Sociol*, 5, 579991. 10.3389/fsoc.2020.579991 33869507 PMC8022438

[cit0068] Trout, L. J., Kramer, C., & Fischer, L. (2018). Social medicine in practice: Realizing the American Indian and Alaska native right to health. *Health and Human Rights Journal*, 20(2), 19–30. PMC6293359 30568399

[cit0069] Vásquez-Vera, H., Fernández, A., Novoa, A. M., Delgado, L., Barcala, J., Macías, C., & Borrell, C. (2019). Our lives in boxes: Perceived community mediators between housing insecurity and health using a PHOTOVOICE approach. *International Journal of Equity in Health*, 18(1), 52. 10.1186/s12939-019-0943-0 PMC6438010 30917833

[cit0070] Wenham, C., Smith, J., & Morgan, R. (2020). COVID-19: The gendered impacts of the outbreak. *The Lancet*, 385(10277), 846–848. 10.1016/S0140-6736(20)30526-2 PMC7124625 32151325

[cit0071] White, M. P., Elliott, L. R., Grellier, J., Economou, T., Bell, S., Bratman, G. N., Cirach, M., Gascon, M., Lima, M. L., Lõhmus, M., Nieuwenhuijsen, M., Ojala, A., Roiko, A., Schultz, P. W., van den Bosch, M., & Fleming, L. E. (2021). Associations between green/blue spaces and mental health across 18 countries. *Scientific Reports*, 11(1). 10.1038/s41598-021-87675-0 PMC8076244 33903601

[cit0072] Wong, A. S., & Kohler, J. C. (2020). Social capital and public health: Responding to the COVID-19 pandemic. *Globalization and Health*, 16(88). 10.1186/s12992-020-00615-x PMC7517063 32977805

